# Roles of miR-223 in Platelet Function and High On-Treatment Platelet Reactivity: A Brief Report and Review

**DOI:** 10.3390/genes16030312

**Published:** 2025-03-06

**Authors:** Shayan Askari, Lawrence E. Goldfinger

**Affiliations:** Cardeza Foundation for Hematologic Research, Department of Medicine, Division of Hematology, Sidney Kimmel Medical College, Thomas Jefferson University, Philadelphia, PA 19107, USA; shayan.askari@students.jefferson.edu

**Keywords:** miR-223, platelet, microRNA, HTPR, clopidogrel, P2Y12

## Abstract

Background: Platelets are highly enriched in microRNAs (miRNAs), which are genomically encoded 19–25 nucleotide non-coding RNAs that target complementary mRNAs through total or near-total base pairing. MiR-223 is among the most abundant miRNAs in human and murine platelets, but despite ongoing investigations in recent years, miR-223 roles in platelet physiology and its putative roles in high on-treatment platelet reactivity (HTPR) remain controversial, as studies showed varying findings. Objectives: In the current hybrid review/report, we aim to compare studies that investigated miR-223 in platelet function and HTPR. Additionally, we briefly report our own findings on murine miR-223-deficient platelets. Methods: We have thoroughly searched the literature and found three studies that investigated the roles of miR-223 in platelet function by utilizing miR-223 global knockout mice, and three studies that explored the association between miR-223 and residual platelet reactivity by measuring miR-223 levels in platelets of patients treated with clopidogrel for cardiac artery disease. We assessed platelet function in response to different agonists and evaluated P2y12 levels in male and female miR-223-deficient platelets. Results: Integrin activation and α granule secretion were similar between WT and KO platelets in response to all agonists in platelets from both female and male mice, although both genotypes showed elevated thrombin response in females compared to males. Conclusions: In all studies, including ours, taken together, miR-233 appears to play a modest role in platelet function and development of HTPR.

## 1. Introduction

Thirty years after their discovery by Ambros and Ruvkun, miRNAs continue to amaze us with their complexity and versatility [1,2]. More than 60% of human coding genes are estimated to be regulated by miRNAs in various cells and tissues [3]; anucleate platelets are no exception. Platelets harbor functional components of RNA-induced silencing machinery such as DICER1 and ARGONAUTE2, capable of processing endogenous miRNAs and exogenous short-inhibitory double-stranded RNAs [4,5,6]. Additionally, suppression of miRNA maturation by megakaryocyte-/platelet-specific Dicer1 deletion in mice increased platelet protein expression and reactivity, underscoring the important contributions of miRNAs to platelet function [7]. We recently demonstrated constitutive translation in peripheral blood platelets which is important for functional response, suggesting further the potential for miRNA modulation of protein expression and functional outcomes in circulating platelets [8]. Initial studies revealed more than 492 different types of mature miRNAs in human platelets [9]. According to two studies utilizing microarray profiling and quantitative PCR, miR-223 is the most abundant miRNA in human platelets [10,11]. The high throughput study by Plé and colleagues, however, estimated miR-223 to be one of the 20 most abundant miRNAs, accounting for just 1.5% of total miRNAs in platelets [9]. The miR-223 gene was first identified in mouse and human samples by the Bartle group in 2003 [12]. Later, to investigate the role of miRNAs in hematopoiesis, Bartle’s team scanned murine hematopoietic tissues, including bone marrow, thymus, and spleen, for miRNA expression by Northern blotting. The study revealed that miR-223, miR-181, and miR-142 were differentially expressed in hematopoietic tissues. Among them, miR-223 was exclusively expressed in bone marrow, with its expression limited to the myeloid lineage [13]. The miR-223 gene locus is located on the X chromosome. Two major mechanisms have been proposed for its transcriptional regulation: (1) NFI-A and C/EBPα compete for binding to the miR-223 promoter, such that binding of C/EBPα promotes upregulation of miR-223 expression which suppresses translation of NFI-A and promotes granulocytic differentiation [14]; (2) transcriptional activation via a conserved 5′-proximal cis-regulatory element downstream of the miR-223 locus which contains binding sites for transcription factor PU.1 and C/EBPα (in RAW264.7 cells and bone marrow-derived human macrophages) [15]. Although miR-223 is enriched in megakaryocytes and platelets and has been studied extensively, the roles of this miRNA in platelet function remain controversial.

## 2. Materials and Methods

Mice deficient in miR-223, B6.Cg-Ptprca Mir223^tm1Fcam^/J, were purchased from The Jackson Laboratory (Farmington, CT, USA). DNA was extracted from ear clippings taken at the time of weaning, typically at 3–5 weeks of age. PCR was performed using GoTaq master mixes (Promega, Madison, WI, USA) according to The Jackson Laboratory recommendations. All mouse protocols and procedures were approved by the Institutional Animal Care and Use Committee of Thomas Jefferson University. This study was carried out in strict accordance with the recommendations in the Guide for the Care and Use of Laboratory Animals of the National Institutes of Health. Blood was collected from the retro-orbital plexus of mice 8–12 weeks of age into EDTA capillary tubes and added to a microfuge tube containing 200 μL 3.8% sodium citrate, followed by the addition of 1 mL Tyrode’s buffer containing 1 mg/mL BSA and 0.1 μg/mL PGE_1_. Samples were centrifuged at 80× *g* for 8 min at RT and platelet-rich supernatants were transferred to 15 mL conical tubes. Apyrase was added at a final concentration of 0.5 U/mL. Samples were subjected to a second centrifugation at 1000× *g* for 10 min at RT. Platelet pellets were gently resuspended in Tyrode’s/BSA and the count was adjusted to 10 × 10^7^ platelets/mL. Platelets were incubated with agonists and 2 mM CaCl_2_ in the presence of saturating amounts of FITC-conjugated α-Cd41 antibodies (clone MWReg30, 133903, BioLegend, San Diego, CA, USA), PE-conjugated α-activated Cd41/61 antibodies (clone Jon/A, 023-2, Emfret, Germany), and/or FITC-conjugated α-Cd62p antibodies (P-selectin, clone RB40.34, 553744, BD Pharmingen, San Diego, CA, USA), or α-P2y12 APC-conjugated antibodies (clone S16007D, BioLegend, San Diego, CA, USA) for 15 min at 37 °C, and analyzed on a BD Accuri C6 Plus flow cytometer. The platelet population was gated based on forward scatter (FSC)/side scatter (SSC) and Cd41 positivity. Beads conjugated with known concentrations of APC (Quantum™ APC MESF, 823, Bangs Laboratories, Fishers, IN, USA) were analyzed according to the manufacturer’s recommendations to obtain the molecular equivalent of soluble fluorophore (MESF).

## 3. Roles of miR-223 as Defined by Global Knockout in Mice

Multiple studies have utilized miR-223 global knockout (KO) mice to determine the roles of miR-223 in blood cell development and function. MiR-223 deletion had no effects on viability and fertility in mice but was associated with neutrophilia driven by increased granulocyte–monocyte progenitors in bone marrow [16]. The -3p arm of miR-223 (miR-223-3p) was reported to target the 3′ untranslated region (3′-UTR) of the (human) P2RY12 mRNA encoding P2Y12 ADP receptor, as evidenced by luciferase assays in HEK and MEG-01 human megakaryocytic cells; a similar miR-223-3p targeting site is evident in the P2ry12 murine mRNA [4,17]. This finding made miR-223 an attractive subject to be studied in the field of platelet biology. An extensive study by Leierseder and colleagues demonstrated that miR-223 ablation had no impact on platelet lifespan, count, or mean volumes, nor were any effects observed on the surface expression of glycoproteins Ibα, IIb, IX, or β1 integrin [18]. Additionally, no differences were observed between KO and wild-type (WT) platelet integrin activation and α granule secretion in response to thrombin or convulxin, or aggregation in response to collagen, ADP, thrombin, or convulxin. However, platelet recovery following antibody depletion was diminished at 72 h in the KO mice compared to WT. The authors deemed this difference to be independent of megakaryopoiesis since miR-223-deficient megakaryocytes demonstrated normal proplatelet formation and ploidy, and bone marrow transplants from KO donors to WT recipients showed similar platelet recovery compared to transplants from WT to WT mice. Contrary to these findings, a later study by Elgheznawy et al. showed that miR-223 KO platelets had a higher percent of maximum aggregation in response to low doses of thrombin and collagen, but demonstrated delayed clot retraction [19]. In the same study, Kindlin3, α2 and β1 integrin, and Factor XIII-A appeared to be increased in miR-223 KO platelets as assessed by Western blot analysis, although α2 and β1 integrin were not altered in the parallel mass spectrometric analysis [19]. A 2017 study by Wang et al. also did not find any differences in platelet aggregation in response to ADP, collagen, or thrombin [20].

**Figure 1:**
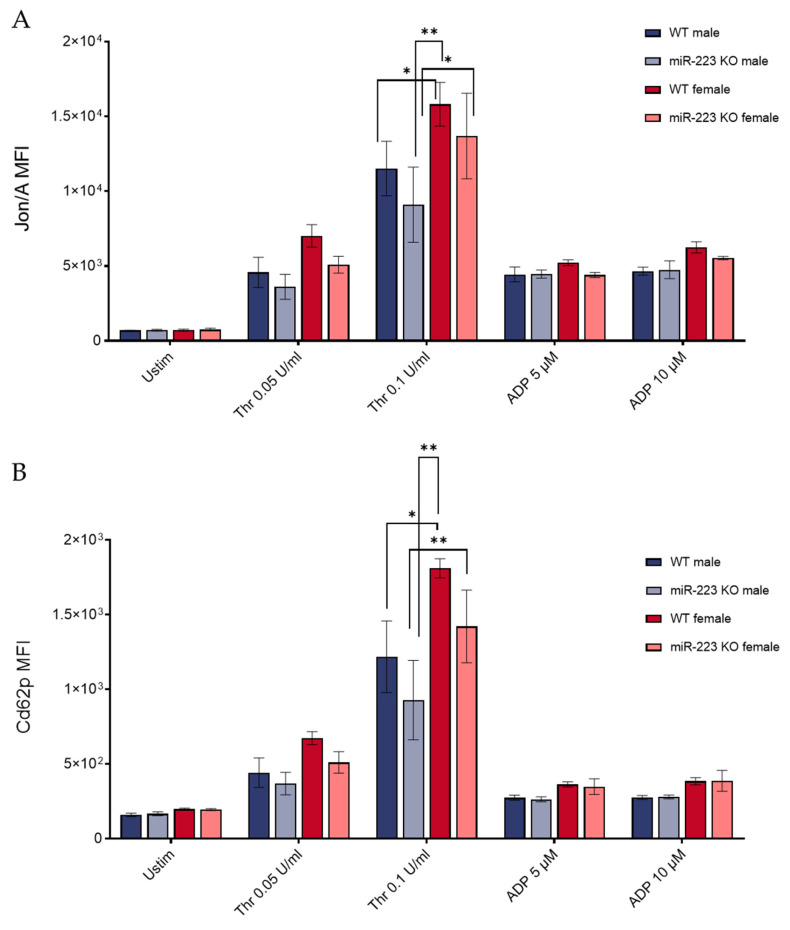
Evaluation of integrin activation and α granule secretion in miR-223 KO platelets. Washed platelets from WT or miR-223 KO mice were subject to agonist stimulation as indicated in the presence of fluorophore-conjugated antibodies against activated (**A**) αIIbβ3 integrin (clone Jon/A) and (**B**) P-selectin (Cd62p) and measured by flow cytometry, as described previously [8]. Tukey multiple comparison analysis performed between groups for each agonist is shown; n = 4, males; n = 3, females; *, *p* < 0.05; **, *p* < 0.01 unlabeled data points showed no significant differences between groups. Thr, thrombin. MFI, mean fluorescence intensities, shown ± SEM.

To our knowledge, all the above studies were restricted to male mice, overlooking potential sex-dependent roles of miR-223 in platelet function and signaling. Furthermore, integrin activation and α granule secretion in response to ADP were not tested, leaving the P2Y12 signaling axis underexplored. To evaluate the roles of miR-223 in platelet activation independently, we assessed αIIbβ3 integrin activation and α granule secretion in female and male miR-223 KO mice. Integrin activation and α granule secretion were similar between WT and KO platelets in response to all agonists in platelets from both female and male mice, although both genotypes showed elevated thrombin response in females compared to males (Figure 1A,B). Thus, overall we did not observe sex-specific differences across genotypes by these measures.

**Figure 2:**
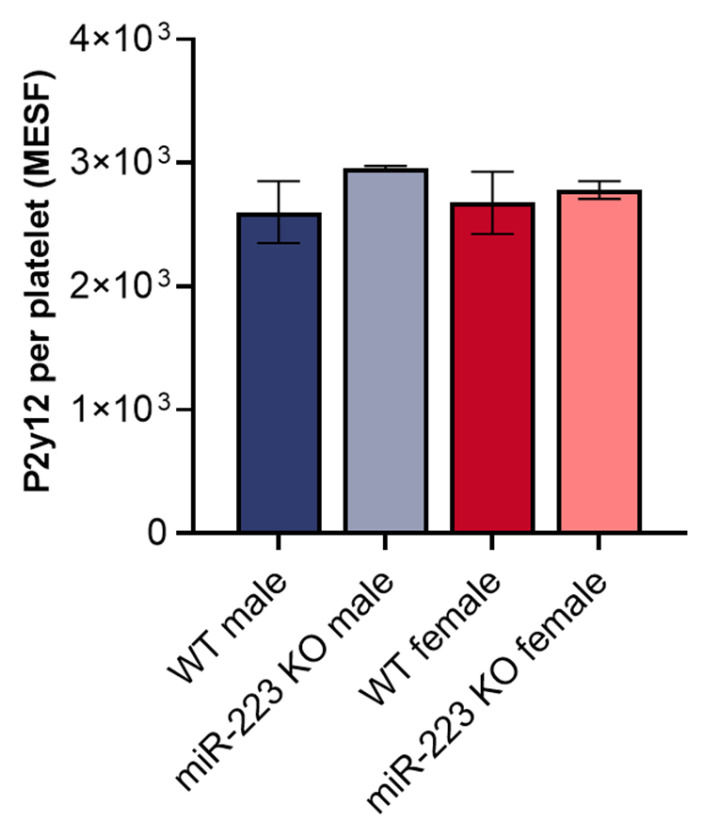
Quantification of P2y12 copy numbers on miR-223 KO platelets. Washed platelets from WT or miR-223 KO mice were labeled with anti-P2y12 fluorophore-conjugated antibodies and measured by flow cytometry. Fluorescent beads with known fluorophore concentrations (Bangs Laboratories) were used to calculate P2y12 MESF. Tukey multiple comparison analysis performed between groups is shown; n = 4, males; n = 3, females; unlabeled data points showed no significant differences between groups. MESF, mean the equivalent of soluble fluorophore, shown ± SEM.

We also used anti-P2Y12 antibodies in conjunction with fluorescence quantification beads to measure surface P2y12 copy numbers in murine platelets. Flow cytometry showed similar levels of surface P2y12 in platelets from male and female WT and KO mice (Figure 2), indicating that although P2ry12 mRNA is a miR-223-3p target, other factors may mitigate potential suppression of P2y12/P2Y12 by miR-223-3p in megakaryocytes and platelets. Considering prior studies and ours together, miR-223 appears not to play a substantial role in platelet P2Y12 expression, signaling, or activation.

Tail tip amputation bleeding time was not different between WT and miR-223 KO mice in two studies [18,19], suggesting that miR-223 is dispensable for hemostasis. Elgheznawy et al. found that KO mice demonstrated faster and larger thrombus formation with a higher rate of emboli in response to carotid arterial injury induced by FeCl3 [19]. Potentiated thrombosis in the FeCl3 injury model, despite delayed clot retraction associated with miR-223 deletion, may relate to increased expression of other proteins besides P2y12 important for platelet function, as mass spectrometry analysis also showed upregulation of Talin1, Alox12, and Rap1b in miR-223 KO platelets. In contrast, in Wang et al., miR-223 KO mice demonstrated a longer time to occlusion in the Rose Bengal carotid injury model. Furthermore, WT mice receiving bone marrow transplants or transfusion of platelets or platelet-derived extracellular vesicles from KO mice showed delayed time to occlusion in the Rose Bengal injury model compared to WT mice receiving bone marrow transplants or transfusions from WT mice [20]. These authors further showed that platelet-derived miR-223 was transferred from platelet-derived extracellular vesicles to endothelial cells, associated with downregulated insulin-like growth factor receptor (IGFR), which has been shown to induce apoptosis in those cells [21]. Intercellular transfer of platelet miRNAs via platelet-derived extracellular vesicles has been shown to have downstream functional effects in target tissues [22,23,24,25,26]. Thus, differential effects on thrombosis as a function of miR-223 deletion likely reflect the roles of exogenous platelet-derived as well as endogenous miR-223 in cells other than platelets, i.e., endothelium, leukocytes [27,28], and vascular smooth muscle cells [29], as well as different experimental approaches.

## 4. MiR-223, High On-Treatment Platelet Reactivity, and Antiplatelets

High on-treatment platelet reactivity (HTPR) is defined as the residual platelet activity present after treatment with antiplatelet medication. HTPR is associated with worse clinical outcomes in patients with cardiovascular complications [30,31]. In recent years, reports of miR-223-3p targeting P2RY12 combined with miR-223 enrichment in platelets led to multiple groups investigating the putative roles of miR-223 in HTPR by assessing its levels in platelets, plasma, or serum. As plasma or serum pools of miRNAs are derived from various cell types, we will focus here exclusively on studies evaluating miR-223 in platelets in the context of HTPR. A study conducted on coronary heart disease patients receiving clopidogrel demonstrated lower levels of platelet miR-223 (-3p or -5p arm not defined) in low-clopidogrel responders, defined by vasodilator-stimulated phosphoprotein (VASP) phosphorylation assay, although no changes were observed in light transmission aggregometry (LTA) in response to 10 μM ADP [32]. Another study by Peng et al. showed an approximately 7-fold decrease in levels of miR-223 (-3p or -5p arm not defined) in leukocyte-depleted platelets in patients with acute coronary syndrome with high on-clopidogrel platelet reactivity (as measured by LTA in response to 20 μm ADP) [33]. In a follow-up study, Liu et al. showed a weak negative correlation (Spearman r = −0.3265) between platelet miR-223-3p and platelet reactivity analyzed by VASP phosphorylation [17]. Although all three of these studies indicate a weak to moderate negative correlation between miR-223 and high platelet on-clopidogrel reactivity, causal relationships and underlying mechanisms remain undefined.

Importantly, clopidogrel is a prodrug primarily metabolized by the CYP2C19 enzyme in the liver, and a strong body of evidence supports the CYP2C19*2 polymorphism, resulting in loss of function playing a significant role in HTPR with both clopidogrel and prasugrel, another P2Y12 antagonist metabolized by CYP2C19 [34,35,36,37,38,39]. In the Peng et al.’s study, when data were adjusted for CYP2C19*2 polymorphism, the association between miR-223 and HTPR was rendered insignificant, supporting a dominant role for this CYP2C19 polymorphism in HTPR with these P2Y12 antagonists [33]. Liu et al. also found that patients with CYP2C19 loss of function polymorphisms (CYP2C19*2, CYP2C19*3) showed significantly higher on-clopidogrel platelet reactivity compared to patients with the reference allele (CYP2C19*1), but correlations with miR-223 levels in these patient cohorts were not established [17]. A comprehensive study by Naggali et al. found no differences in miR-223 levels between hypoactive and hyperactive platelets as classified by maximum aggregation in response to 1.5 μM epinephrine and 4 μM ADP [10]. Taken together, genetic factors such as CYP2C19 polymorphisms regulating drug metabolism rather than P2Y12 expression levels appear to play more prominent roles than miR-233 in HTPR with P2Y12 antagonists.

## 5. Conclusions

Studies in miR-223 knockout mice, including our results, demonstrated varying results, limiting conclusions on the contributions of miR-223 to platelet reactivity in mice. Nonetheless, taken together, miR-223 seems to play at most a minor role in platelet function. Although several studies showed a prothrombic state in the absence of miR-223, thrombosis is a multifaceted phenomenon involving leukocytes and endothelial cells, pointing to important roles for miR-223 in cells other than platelets in gene expression and functional effects modulating thrombosis. Roles for miR-223 in HTPR have been suggested by multiple studies, particularly with clopidogrel. However, HTPR is also a multifactorial phenomenon involving genes not likely to be targeted by miR-223 such as CYP2C19. Moreover, P2Y12 platelet levels do not appear to be affected by altered miR-223 expression. Thus, considering pharmacogenetic and functional studies, it appears unlikely that miR-223 plays a major role in HTPR. In conclusion, caution should be exercised when considering miR-223 as a definitive biomarker for platelet responsiveness to antiplatelet therapy. To establish a precise association between miR-223 and platelet function or HTPR, future studies could take advantage of megakaryocyte-/platelet-specific KO or other tissue-specific approaches to investigate platelet function, as well as further miR-223-focused studies with antiplatelets such as ticagrelor that do not require CYP metabolism.

## Data Availability

All data are provided in the manuscript.

## Abbreviations

The following abbreviations are used in this manuscript:

WT	Wild type (mice)
KO	Knockout (mice)
ADP	Adenosine diphosphate
HTPR	High on-treatment platelet reactivity
LTA	Light transmission aggregometry
VASP	Vasodilator-stimulated phosphoprotein
